# A Highly Efficient Cell Division-Specific CRISPR/Cas9 System Generates Homozygous Mutants for Multiple Genes in *Arabidopsis*

**DOI:** 10.3390/ijms19123925

**Published:** 2018-12-07

**Authors:** Zhengyan Feng, Zhengjing Zhang, Kai Hua, Xifeng Gao, Yanfei Mao, Jose Ramon Botella, Jian-Kang Zhu

**Affiliations:** 1 Shanghai Center for Plant Stress Biology, CAS Center of Excellence in Molecular Plant Sciences, Chinese Academy of Sciences, Shanghai 200032, China; fengzhengyan@sibs.ac.cn (Z.F.); zhangzhengjing@sibs.ac.cn (Z.Z.); huakai@sibs.ac.cn (K.H.); xfgao@psc.ac.cn (X.G.); yfmao@sibs.ac.cn (Y.M.); 2 University of Chinese Academy of Sciences (CAS), Beijing, 100049, China; 3 School of Agriculture and Food Sciences, University of Queensland, Brisbane, QLD 4072, Australia; j.botella@uq.edu.au; 4 Department of Horticulture and Landscape Architecture, Purdue University, West Lafayette, IN 47907, USA

**Keywords:** *Arabidopsis*, cell division-specific Cas9 system, CRISPR/Cas9, multiplex gene editing, Pol III promoter

## Abstract

The CRISPR/Cas9 system has been widely used for targeted genome editing in numerous plant species. In *Arabidopsis*, constitutive promoters usually result in a low efficiency of heritable mutation in the T1 generation. In this work, CRISPR/Cas9 gene editing efficiencies using different promoters to drive *Cas9* expression were evaluated. Expression of *Cas9* under the constitutive CaMV 35S promoter resulted in a 2.3% mutation rate in T1 plants and failed to produce homozygous mutations in the T1 and T2 generations. In contrast, expression of *Cas9* under two cell division-specific promoters, *YAO* and *CDC45*, produced mutation rates of 80.9% to 100% in the T1 generation with nonchimeric mutations in the T1 (4.4–10%) and T2 (32.5–46.1%) generations. The *pCDC45* promoter was used to modify a previously reported multiplex CRISPR/Cas9 system, replacing the original constitutive ubiquitin promoter. The multi-pCDC45-Cas9 system produced higher mutation efficiencies than the multi-pUBQ-Cas9 system in the T1 generation (60.17% vs. 43.71%) as well as higher efficiency of heritable mutations (11.30% vs. 4.31%). Sextuple T2 homozygous mutants were identified from a construct targeting seven individual loci. Our results demonstrate the advantage of using cell division promoters for CRISPR/Cas9 gene editing applications in *Arabidopsis*, especially in multiplex applications.

## 1. Introduction

Traditional reverse genetics methods in plants such as random mutagenesis and T-DNA insertion mutagenesis have limitations, like their inherent random nature, that restrict their usefulness [1,2]. Site-specific genome engineering is a powerful tool to produce targeted mutations based on DNA repair mechanisms triggered by DNA double-strand breaks (DSBs) at specific genomic sites [3]. In recent years, the type II CRISPR/Cas9 system has been widely used for gene editing in animals and plants [4,5,6,7,8]. The CRISPR/Cas9 system is derived from the adaptive immune system of prokaryotes to fight threats by foreign nucleic acids [9]. The endonuclease *Cas9*, guided by the single guide RNA (sgRNA), is capable of cleaving double stranded DNA (dsDNA) at very precise locations which is subsequently repaired by either nonhomologous end-joining (NHEJ) or by homologous recombination (HR) [10]. In the absence of homologous DNA templates, mutations in the target sites, such as deletions, insertions, or base substitutions are generated by the error-prone nonhomologous end joining (NHEJ) pathway [11].

Strong ubiquitous promoters are commonly used to achieve high *Cas9* expression in plant CRISPR/Cas9 cassettes which are usually incorporated into the genome by *Agrobacterium*-mediated or biolistic transformation. In rice and other crop plants, the sgRNA-Cas9 cassettes expressed in embryogenic calli typically produce heritable mutations (homozygous, heterozygous, or bi-allelic) in the T0 generation [5,12]. However, while constitutive *Cas9* expression produces somatic mutations in the majority of *Arabidopsis* T1 plants transformed by the floral dip method, only mutations produced in reproductive cells are transmitted to the next generation [13]. In recent years, germline-specific Cas9 systems have been developed to increase the production of heritable mutations in *Arabidopsis*. The male gametocyte-specific *SPL* and the egg cell-specific *DD45* promoters proved to be highly efficient in the production of T2 heterozygotes [14,15]. In a different approach, the cell division-specific *YAO* promoter produced heritable mutations in the T1 generation [16]. Eid et al. also reported that the application of a meiosis I-specific promoter increased the efficiency of targeted mutagenesis [17]. The available evidence strongly indicates that *Cas9* expression timing and tissue specificity are essential factors affecting the editing efficiency of CRISPR/Cas9 systems.

In yeast, *CDC45* regulates mitotic DNA replication [18]. However, the *Arabidopsis* homolog *AtCDC45* gene is required for initiation of DNA replication and its expression is upregulated at the G1/S transition and in young meiotic flower buds. Transgenic *Arabidopsis* T1 plants harboring an RNA interference construct show partial to complete infertility [19]. Here, we report that, in addition to *YAO*, other cell division specific promoters are capable of generating a high frequency of heritable mutations. We performed a comprehensive study in *Arabidopsis* using constitutive and cell division-specific promoters to drive *Cas9* expression in CRISPR/Cas9 constructs targeting the same locus. Our results show that the *CDC45*-driven *Cas9* system is superior to other previously described systems. The *CDC45* promoter also proved superior to ubiquitous promoters in multiplex CRISPR/Cas9 gene editing systems.

## 2. Results

### 2.1. Cell Division-Specific Promoters Improve the Production of CRISPR/Cas9-Induced Heritable Gene Modifications in Arabidopsis

**Figure 1:**
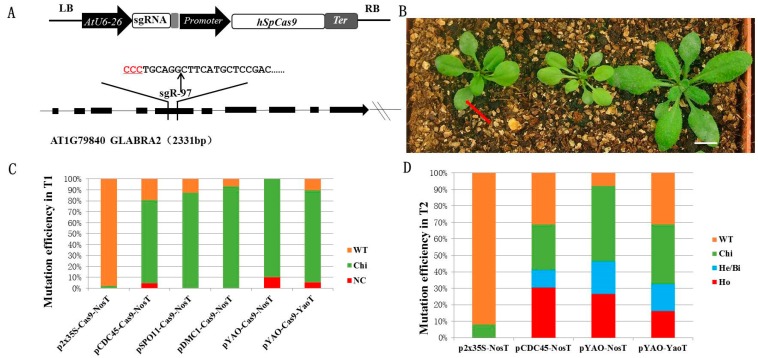
Gene mutagenesis efficiency of cell division specific CRISPR/Cas9 systems in transgenic T1 and T2 generations of *Arabidopsis*. (**A**) Structure of the CRISPR/Cas9 binary vectors and sequence of the target site in *GLABRA2.* The *hSpCas9* gene is driven by the cell division specific promoters *CDC45*, *DMC1*, *SOP11*, and *YAO* as well as the constitutive 2x35S CaMV promoter, while the *AtU6-26* promoter controls transcription of the sgRNA. The sequence of GL2-sgR97 is shown. *hSpCas9*, human codon-optimized *SpCas9*; Ter, Nos or YaoT terminator. The PAM sequence is underlined in red. (**B**) Phenotypes of T1 generation plants transformed with the pCDC45-Cas9 construct. A chimera showing an incomplete glabrous phenotype, a plant showing a uniform glabrous phenotype and a wild type phenotype are shown from left to right. T1 seedlings were transplanted to soil and grown under long-days (16 h light/8 h dark) for two weeks before photographing. Red arrow shows a leaf with a chimeric phenotype. Bar: 1 cm. (**C**) Mutation efficiency in transgenic T1 lines. Approximately 40 individual T1 plants were tested for each construct. NC: non-chimeric mutants (homozygous, bi-allelic, heterozygous); Chi: chimera; WT: wild type. (**D**) Mutation efficiency in transgenic T2 generation. Ho: homozygous; He/Bi: heterozygous/bi-allelic; Chi: chimera; WT: wild type.

Most available CRISPR systems for *Arabidopsis* use constitutive promoters to drive the expression of *Cas9* and, as a consequence, high *Cas9* expression in vegetative tissues results in somatic mutations while heritable mutations are limited to those generated in germline cells. The use of germline specific promoters to direct *Cas9* expression has been recently described as an alternative strategy and several studies have shown that, even though it produces heritable mutations in the T1 generation at low efficiency, it is highly efficient in the generation of heterozygous mutants in the T2 generation [15]. In an attempt to further improve CRISPR efficiency we studied the effect of several cell division-specific promoters to drive *Cas9* expression in *Arabidopsis*. We selected *AtCDC45* (AT3G25100), *AtDMC1* (AT3G22880), and *AtSPO11-1* (AT3G13170) as they are highly expressed in young flower buds and are involved in meiotic divisions [19,20,21]. The promoter regions for each gene (~2 kb) were amplified from *Arabidopsis* genomic DNA by PCR and cloned into the previously described pDD45-GT CRISPR vector replacing the *DD45* promoter [15] (Figure 1A). For comparison purposes the 2x35S promoter and the previously characterized *YAO* (At4G05410) [16,22] promoter were included in the study. While all the above-mentioned CRISPR constructs used the nopaline synthase (Nos) terminator, an additional vector containing the *YAO* promoter upstream of *Cas9* followed by the endogenous *Arabidopsis YAO* terminator was also produced. The *GLABRA2* (*GL2*) gene (AT1G79840), involved in leaf trichome differentiation, was selected as the target since mutations in *GL2* result in a number of easily observable ‘glabrous’ phenotypes such as defects in leaf trichomes [15,23]. The *AtU6-26* promoter was used to transcribe the same sgRNA, designated sgR97, in all constructs (Figure 1A).

The different CRISPR binary vectors were used to transform *Arabidopsis* Col-0 plants using the *Agrobacterium*-mediated floral dip method [24] and a large number T1 transgenic lines (from 30 to 68) were obtained for each construct. The presence or absence of trichomes in leaves was used as a criterion for the initial phenotypic characterization of T1 lines to classify them into three distinct groups consisting of plants showing either a uniform glabrous phenotype in all leaves, a partial chimeric phenotype, or a wild type phenotype (Figure 1B and Supplemental File Table S1). It is important to note that while chimeric phenotypes are relatively easy to identify, the visual classification of a phenotype as ‘uniform glabrous’ does not necessarily exclude the possibility that the phenotype could be produced by multiple chimeric mutations. Using this classification, we observed that 20% to 25% of lines transformed with the pCDC45-Cas9 (Figure 1B) and pYAO-Cas9 constructs (with both terminators) displayed a uniform glabrous phenotype, with an additional 58–70% showing a chimeric phenotype. No glabrous phenotype was observed in plants transformed with p2x35S-Cas9, while pDMC1-Cas9 and pSPO11-Cas9 T1 lines showed chimeric phenotypes in a small number of plants (Supplemental File Table S1). As a more accurate criteria to detect and quantify gene editing efficiency, genomic DNA was isolated from leaf samples of T1 plants, the target site amplified by PCR and the amplicons sequenced to detect mutations [4,25]. Figure 1C and Supplemental File Table S2 show the presence of mutations in 80.9% to 100% of T1 plants transformed with the cell division-specific promoters compared to 2.3% for the p2x35S-Cas9 system. Interestingly, while 87.5% and 93.3% of the T1 plants transformed with pSPO11-Cas9 and pDMC1-Cas9 contained mutations, no homozygous or bi-allelic individuals were identified. In the case of pYAO-Cas9 and pCDC45-Cas9 the frequency of homozygous/bi-allelic individuals ranged from 4.4% to 10% (Figure 1C and Supplemental File Table S2).

We further characterized the phenotypes of a large number of T2 individuals from 10 independent T1 lines for each of the six different CRISPR constructs (between 968 and 1949 per construct). pCDC45-Cas9 proved to be highly efficient with 22.7% of T2 plants showing a uniform glabrous phenotype, while pYAO-Cas9 efficiency was also very high, with NosT and YaoT constructs displaying uniform glabrous phenotypes in 24.5% and 16.1% of the plants, respectively (Supplemental File Table S3). A very small number of uniform glabrous phenotypes were observed in the T2 progeny of pDMC1-Cas9 and pSPO11-Cas9 transgenic lines (3.9% and 0.7%, respectively) while no uniform glabrous plants were observed in the p2x35S-Cas9 lines (Supplemental File Table S3).

Finally, 14-day-old T2 whole-seedlings from eight independent T1 transgenic lines transformed with p2x35S-Cas9, pCDC45-Cas9, or pYAO-Cas9 were characterized by Sanger sequencing of the PCR-amplified target site. The pCDC45-Cas9 construct showed the highest frequency of homozygous T2 mutations at a rate of 30.4% with heterozygous/bi-allelic mutations accounting for an additional 10.6% (Figure 1D and Supplemental File Table S4). The pYAO-Cas9 lines also showed high mutation efficiencies with 26.6% and 16.2% homozygotes for the NosT and YaoT terminators respectively, while no homozygous, bi-allelic, or heterozygous mutations were detected in any of the T2 individuals analyzed for the p2x35S-Cas9 construct.

### 2.2. Development of a Multiplex CDC45 Promoter-Driven CRISPR/Cas9 System

**Figure 2:**
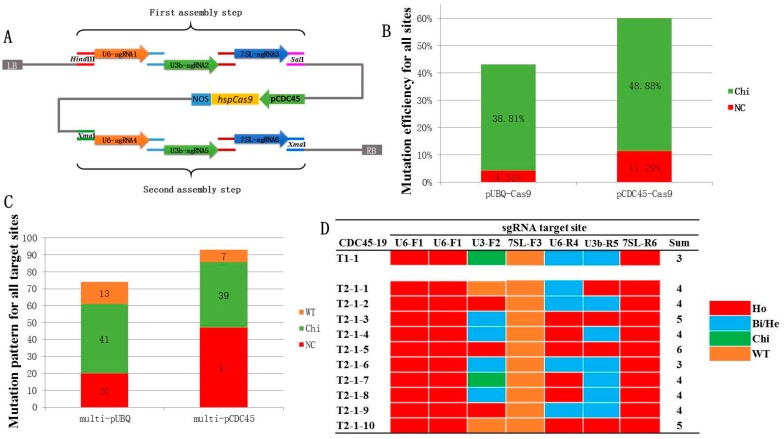
pCDC45-Cas9 system for multiplex gene editing in *Arabidopsis*. (**A**) Schematic diagram of the multi-pCDC45-Cas9 construct. Up to six single guide RNA (sgRNA) fragments with 15 to 20 bp of overlapped sequences were assembled into the pCDC45-Cas9 binary vector by homologous recombination in two steps (3sgRNAs + 3sgRNAs). (**B**) Mutation frequencies and nonchimeric mutation ratios for the multi-pUBQ-Cas9 and multi-pCDC45-Cas9 systems in the T1 generation. For the multi-pUBQ-Cas9 system, 74 target sites were tested by Sanger sequencing while 93 target sites were tested for the multi-pCDC45-Cas9 system. The mutation frequencies were calculated in each target site, and the average of all data used for the two multiplex CRISPR/Cas9 systems. (**C**) Mutation patterns for all target sites for the two multiplex CRISPR/Cas9 systems in the T1 generation. NC: Nonchimeric mutations (homozygous, bi-allelic, heterozygous), Chi: chimera; WT: wild type. (**D**) Zygosity of mutations induced by the CDC45-19 construct in 10 T2 plants from a single T1 line. Sum: The number of target genes with homozygous mutation in individual plants. Ho: homozygous; He: heterozygous; He/Bi: heterozygous/bi-allelic; Chi: chimera; WT: wild type.

Multiplex CRISPR/Cas9 systems have been used to simultaneously edit multiple targets in *Arabidopsis*, greatly simplifying the production of high order mutants [2,26]. To determine whether the *CDC45* promoter could be useful to increase efficiency in multiplex CRISPR/Cas9 we adapted a previously published system that originally used the *Arabidopsis* ubiquitin promoter (*AtUBQ*, AT3G52590) to drive the expression of *Cas9* and three Pol III promoters to control the transcription of up to six different sgRNAs (*AtU6-26*, *AtU3b*, and *At7SL-2*) [26]. For our study the different sgRNA cassettes were assembled into the pCDC45-Cas9 vector in two steps resulting in the multi-pCDC45-Cas9 system. To avoid restriction enzyme-based cloning, the sgRNA cassettes, containing the Pol III promoter, sgRNA target sequence and sgRNA backbone were cloned into the multi-pCDC45-Cas9 vector by homologous recombination (Figure 2A). In our hands, this method is more convenient than the classic restriction enzyme/T4 ligase-based cloning and allowed us to produce a large number of CRISPR constructs containing multiple sgRNA cassettes.

More than 50 *Arabidopsis* genes were selected as target sites for the two multi-CRISPR/Cas9 systems. For the multi-pUBQ-Cas9 system, 22 constructs were prepared containing 2 to 6 sgRNAs each and used to transform wild-type *Arabidopsis* Col-0 plants (Supplemental File Table S5). In total, the multi-pUBQ-Cas9 constructs targeted 74 genomic sites with some sgRNAs having multiple targets. Meanwhile, 93 *Arabidopsis* genomic loci were targeted using 20 multi-pCDC45-Cas9 constructs containing 56 sgRNAs (Supplemental File Table S6). For each target site, approximately 15 independent transgenic T1 lines were tested by PCR amplification of the targeted genomic loci and sequencing of the PCR amplicons. As shown in Figure 2B, the overall average mutation frequency in the 93 loci targeted with the multi-pCDC45-Cas9 system (60.2%) was higher than the efficiency achieved by the multi-pUBQ-Cas9 system (43.7%) (Supplemental File Tables S5 and S6). Nonchimeric (i.e., homozygous, bi-allelic, and heterozygous) mutations were identified in 20 of the 74 target sites for the multi-pUBQ-Cas9 system with frequencies ranging from 4.2% to 37.5% (Figure 2B,C, Supplemental File Table S5 and Figure S1A). For the multi-pCDC45-Cas9 system, nonchimeric mutations were identified in 47 out of the 93 target sites with frequencies of 8.33% to 95.12% (Figure 2B,C, Supplemental File Table S6 and Figure S1B). The overall frequency of nonchimeric mutations obtained with the multi-pCDC45-Cas9 system (11.29%) was close to three times higher than the multi-pUBQ-Cas9 system (4.31%) (Figure 2B).

Finally, we performed a limited analysis of the T2 generation from T1 lines transformed with the CDC45-9, CDC45-15, and CDC45-19 constructs. For each of the three constructs, a single T1 line was selected and the zygosity of 10 individual T2 plants was determined for all the targeted loci (Figure 2D and Supplemental File Figure S2). In the case of the CDC45-19 construct, containing six sgRNAs targeting seven genomic loci, our preliminary analysis of the transgenic CDC45-19 T1 line #1 detected a combination of homozygous mutations (three targets), bi-allelic/heterozygous mutations (two targets), chimeric mutations (one target), and no mutations (one target) (Figure 2D and Supplemental File Table S6). The analysis of 10 individual T2 progeny plants identified different combinations of triple, quadruple, quintuple, and sextuple homozygous mutants (Figure 2D). In the case of CDC45-9 and CDC45-15, targeting three and six genomic loci, respectively, analysis of 10 T2 plants from a single transgenic T1 line unveiled the presence of different combinations of single, double, triple, and quadruple homozygous mutants (Supplemental File Figure S2). These results demonstrate the power of the multi-pCDC45-Cas9 system to generate multiple combinations of homozygous compound mutants in the T1 and T2 generations.

### 2.3. Mutation Frequency of the sgRNA Module Transcribed by Different Pol III Promoters

**Figure 3:**
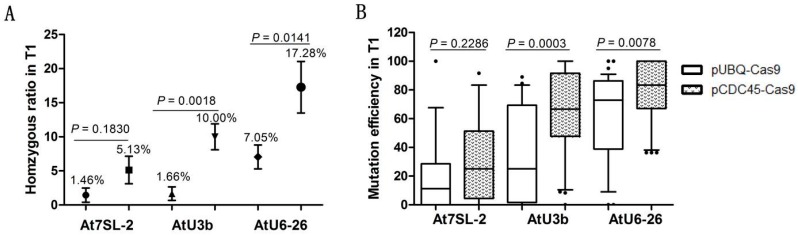
Analysis of mutation frequencies induced by different Pol III promoters. (**A**) Ratio of T1 homozygous mutations for the three Pol III promoters in each of the two multiplex CRISPR/Cas9 systems. (**B**) Mutagenesis efficiency in the T1 generation for the three Pol III promoters in each of the two multiplex CRISPR/Cas9 systems. Data are averages ± SE, Student’s *t*-test were used to value the data.

In this study, the *AtU3b* promoter sequence was modified from the one used by Zhang et al. by deleting two nucleotides and changing the adaptor sequence from “TCACA” to “GGTCG”, so that the transcriptional start site was changed from Adenine “A” to guanine “G”, as is the case for *AtU6-26* and *At7SL-2* promoters [26]. The modified *AtU3b* (*mAtU3b*) was cloned into the multi-pCDC45-Cas9 system, while the nonchanged *AtU3b* was used in the multi-pUBQ-Cas9 system as previous reported [26]. Analysis of the mutation efficiency of all the target genes driven by the three Pol III promoters shows that the *AtU6-26* promoter provided the highest mutation frequency as well as being the most effective in producing homozygous mutations in the T1 generation (Figure 3A,B). The modified *AtU3b* promoter provided comparable overall mutation efficiency to *AtU6-26* while the *At7SL-2* promoter was the least efficient in our experiments. Overall, the multi-pCDC45-Cas9 system proved to be superior to the multi-pUBQ-Cas9 system, regardless of the Pol III promoter used, especially in the production of T1 nonchimeric mutations where ratios for the multi-pCDC45-Cas9 system were between 2.5 to 6 times higher. 

## 3. Discussion

In recent years, the CRISPR/Cas9 system has been widely used for genome editing in plants by genetic transformation with constructs containing the two elements of the system, *Cas9* and sgRNA, usually driven by constitutive promoters. Unlike rice and other crop plants [5,6,7], CRISPR constructs are usually introduced into *Arabidopsis* by the flower dip method resulting in mostly somatic mutations in the first transgenic generation [13]. The use of germline- and cell division-specific promoters to control *Cas9* expression has significantly improved the frequency of heritable mutations and resulted in a detectable (albeit low) frequency of homozygous mutations in the T1 generation [14,15,16]. Aside from the *Cas9* and sgRNA expression levels, the editing efficiency of the CRISPR/Cas9 system is also affected by the features of the target sequence [27,28]. In this study, we compared the mutagenesis efficiency of several cell division-specific promoters versus a strong constitutive promoter (2x35S) in CRISPR/Cas9 constructs targeting the same genomic locus in *Arabidopsis*. The frequency of mutations when *Cas9* was driven by the *YAO* promoter and the *CDC45* meiosis-specific promoter was much higher than the ubiquitous 2x35S promoter in the T1 and T2 generations. However, most of the mutations detected in T1 and T2 plants transformed with pSPO11-Cas9 and pDMC1-Cas9 constructs were somatic mutations, suggesting that *AtSPO11*- and *AtDMC1*-driven expression of *Cas9* is also abundant in vegetative organs. Schmid et al. found that the expression of *AtDMC1* and *AtSPO11* is not restricted to the meiosis phase, but have a high expression level in plant embryos during the development stage, which is in accordance with our results [29]. While this manuscript was in preparation, Xu et al. reported that the MSC system (*Cas9* driven by *DMC1* promoter) induced heritable mutation in T1 generation with high frequency [30]. The differences with our results may be due to the expression levels of *Cas9* affected by short of *AtDMC1* promoter sequence (2172 vs. 3165 bp), and the use of different target sites, which also influence mutation efficiency. In contrast, *CDC45*, whose function is required for the initiation of DNA replication, is expressed predominantly in the G1/S meiosis interphase [19]. The high mutation frequencies observed for the two YAO-Cas9 constructs were consistent with previous results [16]. In our hands, the use of the Nos terminator sequence consistently produced better results than the endogenous *YAO* terminator. Germline-specific-Cas9 systems, such as pDD45-GT, have been reported to induce a high frequency of heterozygous mutations (44.32%) and a lower frequency of homozygotes (6.5%) in the T2 generation [14,15]. In our study the pYAO-Cas9 and pCDC45-Cas9 systems produced a lower frequency of heterozygous mutations (17–30%) but a higher frequency of homozygous mutations (25–49%). Perhaps these differences are due to the fact that *DD45* is preferentially expressed in egg cells while *CDC45* and *YAO* are expressed in gametogenesis allowing the production of mutations in the genomes of both egg and sperm cells during meiotic cell division.

The ability to simultaneously target multiple genes makes the CRISPR/Cas9 system a powerful toolbox for functional genomics and especially useful in the study of gene families. Zhang et al. [26] developed a multiplex CRISPR/Cas9 system that allows the co-expression of six sgRNA modules in a single binary construct in combination with Cas9 driven by the constitutive *Arabidopsis* ubiquitin promoter. We aimed to optimize the original system by using the *CDC45* promoter to increase mutagenesis efficiency and simplifying the cloning of the multiple sgRNA cassettes. In the Zhang et al. [26] multiplex system, the sgRNA cassettes are individually cloned before assembling them into the binary vector using a relatively complex strategy with six multiple adaptive restriction enzymes in two steps. Other strategies such as Golden Gate cloning or Gibson Assembly based on type III restriction enzymes have also been applied to assemble multiplex CRISPR/Cas9 combinations [2,31]. In our present study, the multiple sgRNA modules were cloned into one binary vector based on homologous recombination. The sgRNA modules were amplified with primers containing adaptor sequences to introduce specific homology regions at the 5′ and 3′ ends of each module and then linked into the linearized binary vector by homologous recombination. Given the capacity of homologous recombination-based cloning, up to ten sgRNA modules can be ligated into a single binary construct in two steps with high efficiency. This system does not depend on the availability of multiple restriction endonuclease sites and the ligation of several fragments with compatible ends via T4 ligase, and it is much simpler since it only requires the addition of specific adaptor sequences to the PCR primers.

For a thorough analysis of the *UBQ* and *CDC45* multi-CRISPR/Cas9 systems and to account for the effect of specific features within target sequences, we selected a large number target sites (more than 50 genes) in *Arabidopsis*. We show that the T1 generation mutation efficiency of the multi-pCDC45-Cas9 system (60.17%) is higher than the multi-pUBQ-Cas9 system (43.71%) and it results in a 2.6-fold increase in the production of T1 nonchimeric mutations. Previous reports have shown that multiplex CRISPR/Cas9 systems can generate T0 homozygous mutants for one or more target genes in rice [2,32]. However, in *Arabidopsis* most of the multiple heritable mutants were found in the T2 or T3 generations [26,31]. The use of the *CDC45* promoter in multiplex CRISPR/Cas9 systems can accelerate the production of homozygous mutants in the T1 generation.

Consistent with previous studies, our results show that the mutation efficiency is affected by the promoter controlling the expression of the sgRNA. In our experiments, the *At7SL-2* promoter induced lower mutation efficiencies than the *AtU6-26* and *AtU3b* promoters. We also observed a relative improvement in the efficiency of the *mAtU3b* promoter compared to a previous report [26], perhaps due to the small but significant sequence changes introduced in our system. The position of the *AtU6-26* and *At7SL-2* promoters in the sgRNA cassette has been reported to have no influence on their gene targeting efficiencies in *Arabidopsis* protoplasts [26]. However, Ma et al. [2] found that low sgRNA expression levels result in lower mutation efficiency in *Arabidopsis*. It is therefore possible that the sgRNA mutagenesis activity may be influenced by the expression levels which, in turn, could be related to the relative position in the expression cassette in stable transformants. Our results show that mutation frequencies of sgRNAs in positions F1 and R4 of the multi-binary construct are much higher than those in positions F2, F3, R5, and R6 (Tables S5 and S6). Nevertheless, sgRNAs driven by the same Pol III promoter in different positions within the multi-binary construct of have similar mutation frequencies, denoting the need for additional experiments to answer this question.

Previous studies in plants suggested that a low GC (Guanine-Cytosine) content in the sgRNA sequence might affect gene targeting efficiency, and selection of target sequences with 50% to 70% GC content is desirable [2,28]. However, we did not observe statistically significant differences in mutation efficiencies on target sequences with different GC contents (Supplemental File Figure S3). These results suggest that the range of GC content for the target sites selected in this study (35–65%) has no strong influence on gene editing efficiency. Nevertheless, our work was not designed with this objective in mind and the interpretation of our data should be taken with caution.

In summary, we have compared the mutagenesis efficiencies of several CRISPR systems containing constitutive and cell division-specific promoters driving *Cas9*. Our results demonstrate that the cell division-specific *YAO* and *CDC45* promoters are vastly superior to constitutive promoters in *Arabidopsis* when targeting individual loci or in multiplex applications. 

## 4. Materials and Methods

### 4.1. Plant Transformation and Growth Conditions

*Arabidopsis thaliana* Col-0 ecotype plants were transformed with the different CRISPR/Cas9 binary vectors by the floral dipping method as previously described [33]. For selection of transformants, seeds were sterilized with 5% NaClO for 8 min and plated on 1/2 MS medium with 30 mg/L hygromycin. After 2 weeks, resistant seedlings were transplanted to soil under long-day conditions (16 h light/8 h dark) at 22 °C.

#### Vector Construction

To construct the pCDC45-Cas9, pSPO11-Cas9, and pDMC1-Cas9 plasmids, the promoters of three genes were amplified by PCR from the *Arabidopsis* genome with specific primers (CDC45-F/R SPO11-F/R, and DMC1-F/R) (Supplemental File Table S7). The *AtCDC45* promoter was digested with *Hind*III and *Kpn*I, the *AtSPO11* promoter digested with *NcoI* and *Xho*I, and the *AtDMC1* promoter digested with *Sal*I and *Xho*I, and ligated into the pDD45-GT vector to replace the *DD45* promoter sequence [15]. The *YAO* promoter was PCR-amplified from Col-0 genomic DNA with primers Yao-F and Yao-R (Supplemental File Table S7) and digested with *Xma*I and *Nco*I to replace the 35S promoter in the p35S-Cas9-AtU6 vector [6], producing the pYAO-Cas9 vector. The Yao native transcription terminator was amplified from Col-0 genomic DNA by overlap PCR with two primer pairs to eliminate the *EcoR*I restriction sites in the fragment. The PCR product was digested with *BamH*I and *EcoR*I to replace the Nos terminator in the pYAO-Cas9 vector, producing the pYAO-Cas9-YaoT vector. Two complementary sgRNA nucleotides were synthesized and annealed to generate double-strand DNA with appropriate overhangs on both ends and then inserted into the pAtU6-sgR digested with *Bbs*I [6]. The complete pAtU6-sgRNA cassette was then introduced into the pCDC45-Cas9-1300, pDMC1-Cas9-1300, pYAO-Cas9-1300, pSPO11-Cas9-1300, and p2x35S-Cas9-1300.

For the multi-pUBQ-Cas9 system, the sgRNAs were assembled into the binary vectors as previously described [26]. For the multi-pCDC45-Cas9 system, 18–23 bp sgRNA sequences next to NGG protospacer adjacent motif were synthesized and inserted into the *Bbs*I site in the pAtU6-sgR/pAtU3b-sgR/pAt7SL-sgR vectors. The complete sgRNA cassettes were then amplified with PCR primers containing additional overlap sequences at their ends (15–20 bp) and cloned into the linearized vector using the Exnase MultiS (Homologous recombination ligase, ClonExpress^®^ MultiS One Step Cloning kit, Vazyme, Nanjing, China) in two steps. In the first assembly step, the PCR-amplified sgRNA cassettes (up to three) with the same overlap sequences were tandemly assembled into the pCDC45-Cas9-1300 vector linearized with *Hind*III and *Sal*I (Figure 2A). In a subsequent step, the second set of sgRNA fragments (up to three, making a total of six) with matching overlapping sequences were assembled into the 3sgRNA-pCDC45-Cas9 linearized with *Xma*I. The detailed description of the cloning process for the construction of the binary CRISPR vectors is shown in the methods S1. The primers used in this study are listed in Table S7.

### 4.2. Mutation Detection

Genomic DNA was extracted from rosette leaves of transgenic *Arabidopsis* in the T1 generation by the CTAB method while whole 14-day seedlings were used for DNA extraction in the T2 generation [34]. Genomic regions flanking the target sites were amplified by PCR using primer pairs listed in Table S7. Mutations in target genes were detected by sequencing of the PCR products and the pattern of zygosity was identified by sequence chromatograms. For phenotypic characterization, T2 14-day-old seedlings growing on 1/2 MS medium were observed and the number of mutants with a phenotype similar to *gl2-1* was recorded.

## Supplementary Materials

Supplementary materials can be found at http://www.mdpi.com/1422-0067/19/12/3925/s1.
