# Supplementary material for: A Highly Efficient Cell Division-Specific CRISPR/Cas9 System Generates Homozygous Mutants for Multiple Genes in Arabidopsis
Source: Int J Mol Sci. 2018 Dec 7;19(12):3925. doi: 10.3390/ijms19123925 (PMC6321140; doi:10.3390/ijms19123925)
Supplement: Supplementary file 1 [file ijms-19-03925-s001.pdf]

A highly efficient cell division-specific CRISPR/Cas9 system generates homozygous mutants for multiple genes in *Arabidopsis*

Zhengyan Feng[1], Zhengjiang Zhang[1,2], Kai Hua[1,2], Xifeng Gao[1,2], Yanfei Mao[1], Jose Ramon Botella[3], Jian-Kang Zhu[1,4]

The following Supporting Information is available for this article:

Figure S1. Characterization of mutations in individual multi-CRISPR/Cas9 binary vectors in the T1 generation.

(a) Identification of T1 generation mutations from individual multi-pUBQ-Cas9 binary vectors.

(b) Identification of T1 generation mutations from individual multi-pCDC45-Cas9 binary vectors.

The number of target sites showing non-chimeric mutations, chimeric mutations and Wild-type is indicated. Sequencing results of all loci from a single binary vector are shown in the graph.

Figure S2. Analysis of mutation frequencies induced by the different Pol III promoters.

(a) Zygosity of mutations induced by the CDC45-15 construct in 10 T2 plants from one individual T1 line (#7).

(b) Zygosity of mutations induced by the CDC45-9 construct in 10 T2 plants from one individual T1 line (#7).

Figure S3. Editing efficiency vs. target GC content.

a, Editing efficiencies of all 74 target sites for pUBQ-Cas9 system.

b, Editing efficiencies of all 93 target sites for pCDC-Cas9 system.

Table S1. Phenotypic characterization of T1generation.

Table S2. Mutation analysis of T1generation.

Table S3. Phenotypic analysis of the T2 population segregated from 10 individual T1 lines.

Table S4. Mutation frequencies for different Cas9 systems in the T2 generation.

Table S5. Summary of mutagenesis frequency induced by the multi-AtUBQ-Cas9 system at 74 target sites in the T1 generation.

Table S6. Summary of mutagenesis frequencies induced by the multi-pCDC45-Cas9 system at 93 target sites in the T1 generation.

Table S7. List of primers used in the study.

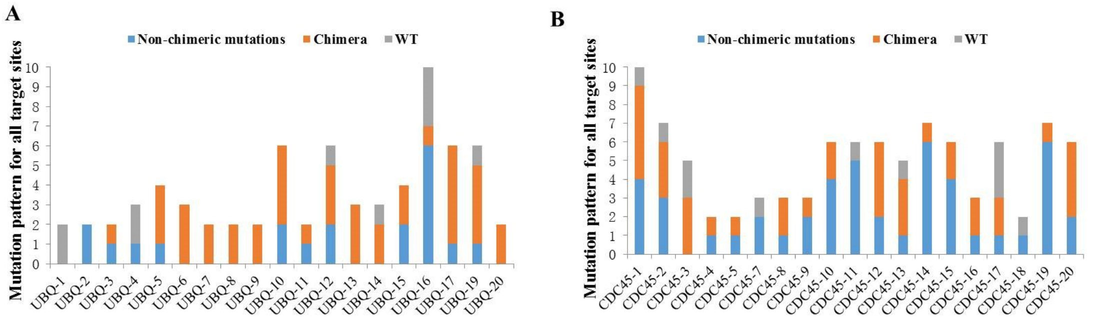

Figure S1. Characterization of mutations in individual multi-CRISPR/Cas9 binary vectors in the T1 generation.

(A) Identification of T1 generation mutations from individual multi-pUBQ-Cas9 binary vectors.

(B) Identification of T1 generation mutations from individual multi-pCDC45-Cas9 binary vectors.

The number of target sites showing non-chimeric mutations, chimeric mutations and Wild-type is indicated. Sequencing results of all loci from a single binary vector are shown in the graph.

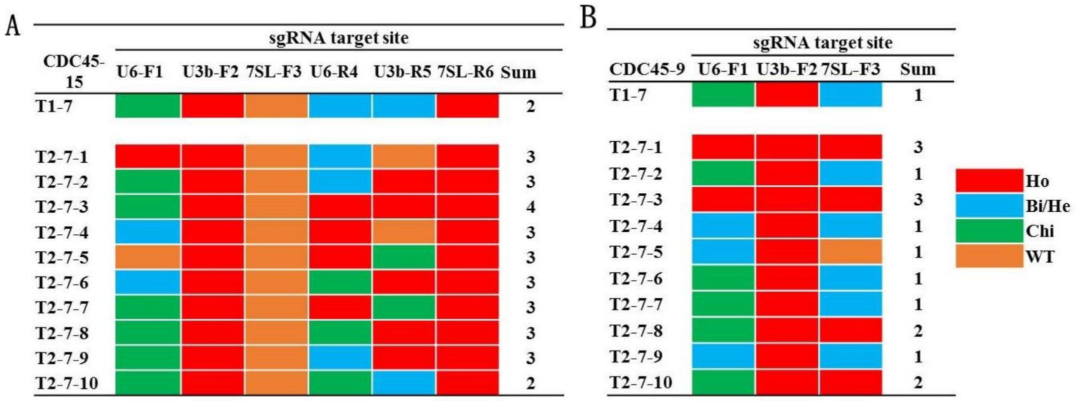

Figure S2. Analysis of mutation frequencies induced by the different Pol III promoters.

(A) Zygosity of mutations induced by the CDC45-15 construct in 10 T2 plants from one individual T1 line (#7).

(B) Zygosity of mutations induced by the CDC45-9 construct in 10 T2 plants from one individual T1 line (#7). Sum: The number of target genes with homozygous mutation in individual plant.

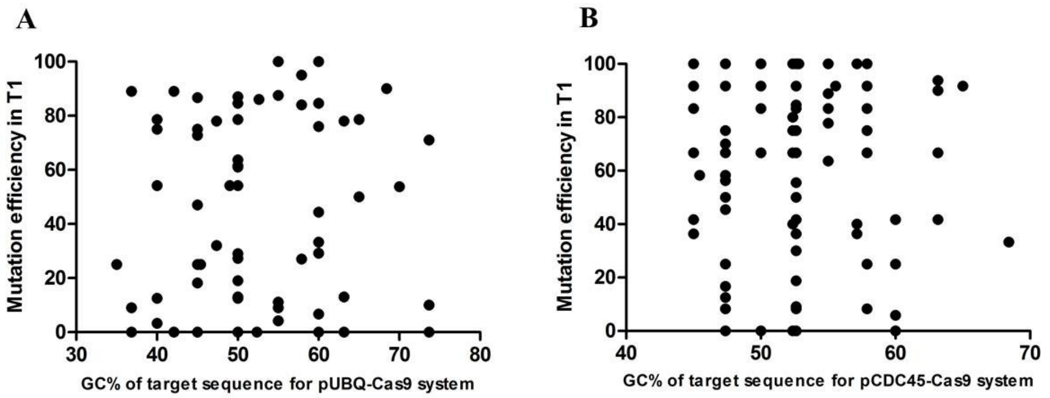

Figure S3. Editing efficiency vs. target GC content.

(A) Editing efficiencies of all 74 target sites for pUBQ-Cas9 system.

(B) Editing efficiencies of all 93 target sites for pCDC-Cas9 system.

Table S1. Phenotypic characterization of T1generation.

	Phenotypic characterization of T1 generation (%)		
	Uniform glabrous phenotype	Chimera	Wild-type
p2x35S-Cas9-sgR97	0	0	100
pCDC45-Cas9-sgR97	23.5	68.1	8.7
pSPO11-Cas9-sgR97	0	5.5	94.5
pDMC1-Cas9-sgR97	0	8.3	91.7
pYAO-Cas9-sgR97-Nos	20	70	10
pYAO-Cas9-sgR97-YaoT	25.4	58	20.3

Table S2. Mutation analysis of T1 generation.

	Zygosity of T1 generation			
	NO. of non-chimeric mutants	NO. of chimera	NO. of wild-type	Total
p2x35S-Cas9-Nos	0	1	42	43
pCDC45-Cas9-Nos	3 (4.4%)	52	13	68
pSPO11-Cas9-Nos	0	56	8	64
pDMC1-Cas9-Nos	0	28	2	30
pYAO-Cas9-Nos	4 (10%)	36	0	40
pYAO-Cas9-YaoT	3 (5.1%)	50	6	59

Table S3. Phenotypic analysis of the T2 population segregated from 10 individual T1 lines.

	Phenotypic segregation of T2 plants					Total
	Uniform glabrous phenotype		Partial glabrous phenotype		Wild-type	
p2x35S-Cas9-Nos	0	0.00%	55	5.68%	913	968
pCDC45-Cas9-Nos	443	22.73%	365	18.73%	1141	1949
pSPO11-Cas9-Nos	13	0.70%	97	5.24%	1740	1850
pDMC1-Cas9-Nos	40	3.94%	108	10.63%	868	1016
pYAO-Cas9-Nos	302	24.47%	250	20.26%	682	1234
pYAO-Cas9-YaoT	217	16.13%	197	14.65%	931	1345

The T2 seeds were sown on 1/2 MS medium and evaluated after 2 weeks.

Table S4. Mutation frequencies for different Cas9 systems in the T2 generation.

	NO.of homozygotes	NO.of heterozygotes or Bi-allele	NO.of chimera	NO.of wild-type	NO.of total plants
2x35S-Cas9-Nos	0	0	7	81	88
pCDC45-Cas9-Nos	49	17	45	50	161
pYAO-Cas9-Nos	41	30	71	12	154
pYAO-Cas9-yaoT	25	25	56	48	154

Table S5. Summary of mutagenesis frequency induced by the multi-AtUBQ-Cas9 system at 74 target sites in the T1 generation.

Constructs	NO.of sgRNA in a vector	relative positon to Cas9 in vector	Target gene	promoter	Target Sequence	PAM sequence	length of Target	GC content	mutation efficiency in T1	homozygous ratio(sequencing in T1)
UBQ-1	2	F1	AT2G30350	AtU6	GTCGGTGAGGATGGAGTCAC	CGGAAA	20	60	0	0
		R4		AtU6	CGGAGAATAAGGCAGCACAA	TGGAGA	20	50	0	0
UBQ-2	2	F1	AT2G30350	AtU6	TCACGGTATCACTATCATGA	TGGGTA	20	40	78.6	25
		R4		AtU6	GACGACTGAGGTTGATGTAG	AGGTGA	20	50	78.6	25
UBQ-3	2	F1	AT5G53800	AtU6	AACGATTCCGGCTCTGAATC	TGGTTT	20	50	13	0
		R4		AtU6	AACGTCCAGAGTTCACAGCA	TGGTTG	20	50	87	21.7
UBQ-4	3	F1	AT1G48410	AtU6	GAGCCAGAGACATCAGACAG	TGGCTC	20	55	100	0
		F2	AT1G69440	AtU3B	TGATAAGGCTTGTGGAGAAG	AGGTGT	20	45	0	0
		F3	AT5G43810	At7SL	CACCGTCAAAGAATCGTAGC	CGGAGG	20	50	0	0
UBQ-5	3	F1	AT1G31280	AtU6	GCGTGATGTGTTGCAAGGAA	TGGATG	20	50	61.5	0
			AT1G31290			TGGATG	20	50	84.6	0
		F2	AT2G27040	AtU3B	GGGGCCAGTTCGAGTTCCTA	TGGCTC	20	60	84.6	0
		F3	AT2G32940	At7SL	AGACGTGGGGTTGGAACCAC	AGGCAA	20	60	100	15
UBQ-6	6	R4	AT2G27880	AtU6	GTTTCCGCCGGTAGAGGTCG	TGGAAA	20	65	50	0
		R5	AT5G21030	AtU3B	CGCCGTGGCAACGGTTCCAA	AGGACA	20	65	78.6	0
		R6	AT5G21150	At7SL	CCTCGAGGCAGCGGCTCCAA	AGGACA	20	70	53.8	0
UBQ-7	2	F1	AT1G69120	AtU6	GGGTAGGGTTCAATTGAAG	AGGATA	19	47.37	32	0
		R4		AtU6	TGAAGTTACCAAGAATCAG	TGGAGT	19	36.84	9	0
UBQ-8	2	F1	AT5G13930	AtU6	AGAGAGCTGATGGACCTGC	AGGCAT	19	57.9	84	0
		R4		AtU6	AGGCGACAAGTCGACAATT	CGGAAA	19	47.37	78	0
UBQ-9	2	F1	AT4G18480	AtU6	CCCCCATTTGCTTCAGGCC	AGGTAA	19	60	76	0

		R4		AtU6	GACATTCATAACAGAGACA	TGGTAA	19	36.84	89	0
UBQ-10	6	F1	AT1G70790	AtU6	GTCAGTTGTTCGTTCCACAC	TGGATT	20	50	54.2	4.2
		F2	AT1G66360	AtU3B	CCCATACGGACAATGACATA	AGGATC	20	45	25	0
		F3	AT1G23140	At7SL	CGTCGTAACCATGGGTTCTC	AGGTAT	20	55	4.2	0
		R4	AT1G70810	AtU6	CTGCAACCCTGAGTGGAACG	AGGAAT	20	60	33.3	8.3
		R5	AT2G01540	AtU3B	GCTGCTACGATGGTCGCGAA	TGGCGA	20	60	29.2	0
		R6	AT1G73580	At7SL	GCTGCTGGAAACATCACGTA	CGGCGA	20	50	12.5	0
UBQ-11	2	F	AT1G70800	AtU6	CGTCATAACCATGGGTCCAC	AGGTAC	20	55	87.5	20.8
		R	AT1G48590	AtU6	CTCCGTATTCGCATCAAACG	CGGCGT	20	50	54.2	0
UBQ-12	6	F1	AT2G46440	AtU6	GACCGTCTTGTTAGCTTGCG	TGGTTG	20	55	9	0
		R4	AT2G46450	AtU6	CGTTCTCAAGCATCTTTGAG	TGGTTG	20	45	72.8	27.3
		F2	AT2G46430	AtU3B	TCTTCTAGTGGTTTCGGCAG	AGGAGA	20	50	63.7	18.2
		R5	AT2G46430	AtU3B	GTAACTGAAACTGCTTGGGC	TGGTGC	20	50	27.3	0
		F3	AT1G01340	At7SL	TAGAACGAGAAGACAGATGC	TGGC	20	45	18.2	0
		R6	AT4G01010	At7SL	TTCTTCTACTGCTTCTGGTG	GGGT	20	45	0	0
UBQ-13	3	F1	AT4G25470	AtU6	TCGCCGCCATAGCTCTCCG	TGGCAG	19	68.42	90	0
		F2	AT4G25480	AtU3B	GGCTTGGAGACTCCGAATCC	CGGAAT	20	60	6.7	0
		F3	AT4G25490	AtAt7SL	GGACTTTCCAAACCGCTGAGA	TGGCAG	21	52.38	0	0
UBQ-14	3	F1	AT4G25470	AtU6	TCGCCGCCATAGCTCTCCG	TGGCAG	19	68.42	90	0
		F2	AT4G25490	AtU3B	TCGCTGCATTAGCCCTCCG	TGGCCG	19	63.16	0	0
		F3	AT4G25480	At7SL	GCGGCGGCTGAAGCTGCGT	TGGCGT	19	73.68	10	0
UBQ-15	4	F1	AT4G36820	AtU6	GCCACGATTGATCCAGAGCC	TGGAGA	20	60	44.4	11.1
		F2	AT2G23790	AtU3B	CAGTGCTATGATTAGGCCGG	CGGAGA	20	55	11.1	0
		R1	AT1G09570	AtU6	AGGACATGGCTTGGTCACGG	TGGATT	20	60	100	37.5
		R2	AT5G42610	AtU3B	CAGTACATGACTTCACCTAA	TGGTCC	20	40	12.5	0

UBQ-16	6	F1	AT4G22120	AtU6	TATCTCCGGATTTACTGGCT	CGGGTA	20	45	75	0
			AT4G04340			GGGGTA	20	45	75	8.3
		R4	AT1G62320	AtU6	ATCATCGTTAGAGAGAAGAG	CGGCTT	20	49	54.2	8.3
			AT1G11960			CGGCTT	20	40	54.2	8.3
		F2	AT4G15430	AtU3B	TTCCCTAAATGGTATCTCAA	GGGTTT	20	35	25	8.3
			AT3G21620			GGGCTT	20	35	25	8.3
		R5	AT4G15430	AtU3B	TTTTGGGTGCATTTGTGTA	TGGCTT	19	36.84	0	0
			AT3G21620			TGGCTT	19	36.84	0	0
		R6	AT4G02900	At7SL	TCAGGCATTTGAAGAGCAGC	AGGCAT	20	50	29	8.3
		F3	AT1G32090	At7SL	CGCTCCGACAGAACTCTCGT	CGGAAA	20	60	0	0
UBQ-17	6	F1	AT5G46790	AtU6	CGGTGGTGAACATAGGCTG	AGGAAT	19	57.9	95	28.6
		F2	AT4G17870	AtU3B	AGTTGGTATGTGTGGAACT	CGGCGA	19	42.1	89	0
		F3	AT2G38310	At7SL	GCGGAGACGGCGATAACGT	TGGTAG	19	63.16	13	0
		R4	AT2G26040	AtU6	CGCTCCGGCCTCCGTGGTT	TGGCCT	19	73.68	71	0
		R5	AT5G53160	AtU3B	TTTGTCGTATCCGACGCGG	AGGTAT	19	57.9	27	0
		R6	AT5G05440	At7SL	AGCCTCACGATCAGACCGA	TGGTCC	19	57.9	27	0
UBQ-18	6	F2	AT5G53160	AtU3B	ACATCATAAGCATGAGCTTG	TGGATA	20	40	3.3	0
UBQ-19	6	F1	AT4G01026	AtU6	ACACTGGTTCTCTCTGCAG	TGGTGA	19	52.63	86	13.2
		F2	AT1G73000	AtU3B	GCACACCGTGTAGACGCAC	CGGCAC	19	63.16	78	0
		F3	AT2G40330	At7SL	GTGAGCCTCACGCGCGGGA	TGGCTG	19	73.68	0	0
		R4	AT1G01360	AtU6	TCGAGACGGTGCAATACGTA	CGGACG	20	50	61	0
		R5	AT5G45860	AtU3B	ATGCTCCACTATCTCTAGTT	TGGTCA	20	40	75	0
		R6	AT5G45870	At7SL	ATGCTCCGTTACCTCTAGTA	TGGTCG	20	45	47	0
UBQ-20	2	F1	AT4G27920	AtU6	GAGGTTGGTAGCGTAAGAGAAG	TGGATT	22	50	19	0
		R4	AT4G18620	AtU6	TGAAGCACCATTACCACTAGTG	TGGTCC	22	45.4	25	0

UBQ-21	6	R5	AT2G40330	AtU3B	TCTGAAACTGTATCGACGT	TGGCAT	19	42.11	0	0
		R6	AT2G40330	At7SL	GAGCTTTCCCACACGCACG	TGGTTG	19	63.16	0	0
UBQ-22	3	F1	AT4G27920	AtU6	ATCAGTAGGTGTGTGGTACA	AGGTAA	20	45	86.7	13.3

22 binary vectors with 70 sgRNAs were introduced into *Arabidopsis* by the flower dip method. The PAM sequences are shown in red.

Table S6. Summary of mutagenesis frequencies induced by the multi-pCDC45-Cas9 system at 93 target sites in the T1 generation.

Constructs	No.the sgRNA in a vector	relative positon to Cas9 in vector	Target gene	promoter	Target sequence	PAM sequence	length of Target	GC content	mutation efficiency in T1(%)	homozygous ratio
CDC45-1	6	F1	At3g22490	AtU6	GGCTTATCAGCGAGTTCAC	CGGAG	19	52.63	83.33	0.00
			At3g22500		GGGCTTATCAGCGAGTTCAC	CGGAG	20	55.00	83.33	0.00
		F2	At3g22490	AtU3b	ACTCACCCTGGTGGTGTAG	CGGCT	19	57.89	66.67	8.33
			At3g22500			CGGCT	19	57.89	83.33	8.33
		F3	At1g03120	At7SL	CACGGCCACATGATCAATA	CGGCA	19	47.37	8.33	0.00
		R4	At5g53260	AtU6	CGGATTCAGCAACACCTCC	AGGAA	19	57.89	91.67	8.33
			At5g53270			AGGAA	19	57.89	100.00	0.00
		R5	At5g53260	AtU3b	GTGGCTGCCATAAAAGAGG	TGGAA	19	52.63	100.00	8.33
			At5g53270		TGGCTGCCATAAAAGAGG	TGGAA	18	55.56	91.67	0.00
		R6	At5g27980	At7SL	AAATCAACCTGTTCACCGG	AGGCC	19	47.37	0.00	0.00
CDC45-2	6	F1	At1g20440	AtU6	ACAGAGGAAGAAGAGCTGT	TGGAT	19	47.37	91.67	33.33
			At1g20450			TGGAT	19	47.37	75.00	25.00
		F2	At1g76180	AtU3b	GCTCAAACTCTGAAGCGAT	CGGAG	19	47.37	75.00	0.00
		F3	At4g38410	At7SL	GAGAATGCCCGTGTAACCA	AGGAG	19	52.63	41.67	0.00
		R1	At5g66400	AtU6	GTACTCGTCATACTGCTGC	TGGAT	19	52.63	75.00	0.00
		R2	At3g50980	AtU3b	GAGTAGCTCTAGCTCTAGCT	CGGTA	20	50.00	100.00	33.33
		R3	At2g21490	At7SL	CATCAGTCAGGTCGACAAT	TGGGT	19	47.37	0.00	0.00
CDC45-3	6	F2	At4g39130	AtU3b	TGGTGAGGAGTATTGGTGG	TGGTG	19	52.63	91.67	0.00
		F3	At1g54410	At7SL	CTTGTTGCCTCCTCCAATG	TGGAG	19	52.63	0.00	0.00

		R4	At2g23110	AtU6	TGGGGTCTTGATAGACTCG	AGGTC	19	52.63	36.36	0.00
		R5	At2g23120	AtU3b	GATAGCCCTTACATGGCGA	CGGGT	19	52.63	9.09	0.00
		R6	At2g33690	At7SL	TATGGAACTTCCGGTCACC	AGGAC	19	52.63	0.00	0.00
**CDC45-4**	**2**	F1	At2g41260	AtU6	CTCACTCGACATAGTTGAC	TGGTT	19	47.37	100.00	65.85
		F2	At2g41280	AtU3b	CTCATGTCTCTCGTTCTCG	TGGCT	19	52.63	18.75	0.00
**CDC45-5**	**2**	F1	At2g41260	AtU6	CTCAAGTCTCTCGTTCTCT	TGGCT	19	47.37	56.25	0.00
		F2	At2g41280	AtU3b	TGTTGCCGCAGTCAAGCCG	AGGCC	19	63.16	93.75	6.25
**CDC45-6**	**3**	F2	At2g23120	AtU3b	GATAGCCCTTACATGGCGA	CGGGT	19	52.63	30.00	0.00
**CDC45-7**	**3**	F1	At5g06760	AtU6	GAAACGCGTCAGCACAACG	CGGCC	19	57.89	75.00	25.00
		F2	At2g35300	AtU3b	TCTGATGGGTTGTACTGTG	AGGGA	19	47.37	58.33	16.67
		F3	At1g32560	At7SL	GATATGGCTAGTACAGCCA	AGGAG	19	47.37	50.00	0.00
**CDC45-8**	**3**	F1	At1g01470	AtU6	CCTACAACAGGAAGGTCGA	TGGTT	19	52.63	84.62	15.38
		F2	At2g44060	AtU3b	GACATTGGTGAGAAACTCG	AGGGA	19	47.37	12.50	0.00
		F3	At2g46140	At7SL	GCTGGCGAATATTCCGACGC	CGGAA	20	60.00	5.88	0.00
**CDC45-9**	**3**	F1	At4g02380	AtU6	GACTTACCGGAAGATAGCAT	TGGAG	20	45.00	83.33	0.00
		F2	At1g02820	AtU3b	GCTTCAAGCGAGAAGGCACCA	TGGGT	21	57.14	100.00	36.36
		F3	At3g53770	At7SL	GTAGTCAAGAGAAGCCATCG	TGGGC	20	50.00	83.33	33.33
**CDC45-10**	**6**	F1	At4g02380	AtU6	GACTTACCGGAAGATAGCAT	TGGAG	20	45.00	91.67	8.33
		F2	At1g02820	AtU3b	GCTTCAAGCGAGAAGGCACCA	TGGGT	21	57.14	100.00	9.09
		F3	At3g53770	At7SL	GTAGTCAAGAGAAGCCATCG	TGGGC	20	50.00	91.67	25.00
		R4	At4g15910	AtU6	TGGCCGCTCGTTCACTCTC	CGGTG	19	63.16	90.00	0.00
		R5	At3g51810	AtU3b	GCTTGATGAGAAGGCGAAGCA	AGGAG	21	52.38	75.00	8.33
		R6	At2g40170	At7SL	CCCGTACCACCTGGCACGA	CGGTC	19	68.42	33.33	0.00
**CDC45-11**	6	F1	At5g06760	AtU6	GAAACGCGTCAGCACAACG	CGGCC	19	57.89	75.00	8.33
		F2	At2g35300	AtU3b	TCTGATGGGTTGTACTGTG	AGGGA	19	47.37	50.00	16.67

		F3	At1g32560	At7SL	GATATGGCTAGTACAGCCA	AGGAG	19	47.37	16.67	16.67
		R4	At1g01470	AtU6	CCTACAACAGGAAGGTCGA	TGGTT	19	52.63	75.00	8.33
		R5	At2g44060	AtU3b	GACATTGGTGAGAAACTCG	AGGGA	19	47.37	8.33	8.33
		R6	At2g46140	At7SL	GCTGGCGAATATTCCGACGC	CGGAA	20	60.00	0.00	0.00
CDC45-12	6	F1	At1g72100	AtU6	GGCAATAGAAAGGCAAGTGA	CGGAA	20	45.00	100.00	60.00
		F2	At2g03850	AtU3b	GATCAGTCGAGGCAAGAGAAG	CGGCG	21	52.38	80.00	10.00
		F3	At2g42540	At7SL	GGAGCTGTTCTCACTGGTA	TGGCT	19	52.63	55.56	0.00
		R4	At2g03740	AtU6	GCACCTCAGCGCAAGAAGTCA	TGGGT	21	57.14	40.00	0.00
		R5	At2g42530	AtU3b	GGTGTTGGTACCGTCAGAGT	TGGCC	20	55.00	88.89	0.00
		R6	At2g42560	At7SL	GGTGACGGAGAGAGAAGTTC	AGGTG	20	55.00	77.78	0.00
CDC45-13	6	F1	At4g21020	AtU6	TAGATAACGCTAGAGACTCG	AGGGC	20	45.00	36.36	0.00
		F2	At4g13230	AtU3b	GTCTGTGACAAAGCCACAG	AGGCT	19	52.63	91.67	16.67
		F3	At2g36640	At7SL	ATTGTGACATCAGCACCAC	CGGAT	19	47.37	25.00	0.00
		R5	At3g53040	AtU3b	GCTGCAGCCTCAGCTCTCT	CGGCC	19	63.16	41.67	0.00
		R6	At3g17520	At7SL	GTTGTGTCCAAGCTACGATCG	AGGAA	21	52.38	0.00	0.00
CDC45-14	6	F1	At1g20440	AtU6	ACAGAGGAAGAAGAGCTGT	TGGAT	19	47.37	100.00	16.67
			At1g20450			TGGAT	19	47.37	75.00	16.67
		F2	At1g76180	AtU3b	GCTCAAACTCTGAAGCGAT	CGGAG	19	47.37	66.67	25.00
		F3	At2g36640	At7SL	ATTGTGACATCAGCACCAC	CGGAT	19	47.37	16.67	0.00
		R4	At3g53040	AtU6	GATAGGCTCGGTGATGAAGG	CGGTG	20	55.00	100.00	25.00
		R5	At2g44060	AtU3b	GACATTGGTGAGAAACTCG	AGGGA	19	47.37	45.45	18.18
		R6	At2g42560	At7SL	GGTGACGGAGAGAGAAGTTC	AGGTG	20	55.00	83.33	25.00
CDC45-15	6	F1	At4g21020	AtU6	TAGATAACGCTAGAGACTCG	AGGGC	20	45.00	41.67	16.67
		F2	At4g15910	AtU3b	CGCAGAATGTAACAGCAGC	AGGAT	19	52.63	66.67	16.67
		F3	At4g02380	At7SL	AGACGTGGTTATGCGGCCA	CGGCG	19	57.89	25.00	0.00

		R4	At5g44310	AtU6	GAGAGACTCGAGGGCCGACT	TGGCA	20	65.00	91.67	58.33
		R5	At4g02380	AtU3b	GACTTACCGGAAGATAGCAT	TGGAG	20	45.00	66.67	0.00
		R6	At3g53770	At7SL	GTAGTCAAGAGAAGCCATCG	TGGGC	20	50.00	66.67	25.00
**CDC45-16**	**5**	F1	At2g03740	AtU6	GCACCTCAGCGCAAGAAGTCA	TGGGT	21	57.14	36.36	0.00
		F2	At2g03850	AtU3b	GATCAGTCGAGGCAAGAGAAG	CGGCG	21	52.38	66.67	8.33
		R5	At2g42530	AtU3b	GGTGTTGGTACCGTCAGAGT	TGGCC	20	55.00	63.64	0.00
**CDC45-17**	**6**	F1	At2g18340	AtU6	GGGGAAATGGTTAGAGTCGA	CGGCG	20	50.00	100.00	85.71
		F3	At3g17520	At7SL	GTTGTGTCCAAGCTACGATCG	AGGAA	21	52.38	40.00	0.00
		R4	At4g36600	AtU6	GAACGGAGACAGCAGAGGATA	TGGTT	21	52.38	100.00	0.00
**CDC45-18**	**2**	F1	At1g72110	AtU6	GGCAATAGAAAGGCAAGTGA	CGGAA	20	45.00	100.00	45.45
		F2		AtU3b	GATCACAAAGGTGATGAGGGTC	TGGAA	22	50.00	0.00	0.00
**CDC45-19**	**6**	F1	At1g20440	AtU6	ACAGAGGAAGAAGAGCTGT	TGGAT	19	47.37	50.00	20.00
			At1g20450			TGGAT	19	47.37	70.00	20.00
		F2	At1g01470	AtU3b	CACACCAACGTCTCGAGCC	AGGTT	19	63.16	66.67	33.33
		F3	At1g54410	At7SL	CTTGTTGCCTCCTCCAATG	TGGAG	19	52.63	8.33	0.00
		R4	At4g02380	AtU6	GACTTACCGGAAGATAGCAT	TGGAG	20	45.00	91.67	16.67
		R5	At4g15910	AtU3b	CGCAGAATGTAACAGCAGC	AGGAT	19	52.63	75.00	25.00
		R6	At5g66400	At7SL	GGTGGCCAAGGATACGGAAC	CGGGA	20	60.00	25.00	8.33
**CDC45-20**	**6**	F1	At5g06760	AtU6	GAAACGCGTCAGCACAACG	CGGCC	19	57.89	100.00	8.33
		F2	At4g15910	AtU3b	CGCAGAATGTAACAGCAGC	AGGAT	19	52.83	100.00	16.67
		F3	At5g66400	At7SL	GGTGGCCAAGGATACGGAAC	CGGGA	20	60.00	41.67	0.00
		R4	At1g52690	AtU6	GACAAGACAGGAAGCTACATGT	CGGAA	22	45.45	58.33	0.00
		R5	At2g23120	AtU3b	GATAGCCCTTACATGGCGA	CGGGT	19	52.63	50.00	0.00
		R6	At4g02380	At7SL	AGACGTGGTTATGCGGCCA	CGGCG	19	57.89	8.33	0.00

20 binary vectors with 56 sgRNAs were introduced into *Arabidopsis* by the flower dip method. The PAM sequences are shown in red.

Table S7. List of primers used in the study.

YAO-F	TCAGCCCGGGACAAATAGAGGTAGGGGGAGAGTT
YAO-R	TGAGCCATGGTCTTCTCTCTCTCACTCCCTCTTAG
CDC45-F	TTTAAGCTTCTCCTGATGATAAAGGTGGGAG
CDC45-R	TTTCTCGAGTTCCGTGAAATTGAATCACCC
DMC1-F	AAAGTCGACCAGGGAATGTTCCAATATAAGAC
DMC1-R	TTTCTCGAGTTTCTCGCTCTAAGAGTCTCTAAG
SPO11-F	CCATGGGCATTTGCAGCTCGTCTGG
SPO11-R	CTCGAGCTCTTTCGAGTTTCAAAACTGAAA
VU6-1F	AACGACGGCCAGTGCCAAGCTTCATTCGGAGTTTTTGTATCTTG
VU6-R	GTTATCCATCACAGGCTCGAGCCATTTGTCTGCAGAATTGGC
VU6-1R	CTTTATCATCAGGAGGTCGACCCATTTGTCTGCAGAATTGGC
VU3-F	CTCGAGCCTGTGATGGATAAC
VU3-R	GCCAATTTACCAGCATCTAGACCATTTGTCTGCAGAATTGGC
VU3-1R	CTTTATCATCAGGAGGTCGACCCATTTGTCTGCAGAATTGGC
V7SL-F	TCTAGATGCTGGTAAATTGGC
V7SL-1R	CTTTATCATCAGGAGGTCGACGCCATTTGTCTGCAGAATTGGC
VU6-2F	ATGTTACTAGATCGGCCCGGGCATTCGGAGTTTTTGTATC
VU3-2R	AATTCGAGCTCGGTACCCGGGCCATTTGTCTGCAGAATTGGC
V7SL-2R	AATTCGAGCTCGGTACCCGGGGCCATTTGTCTGCAGAATTG
1300-F	GGCGATTAAGTTGGGTAACG
CDC45-R	GACATTGGTCTAGAGTTACAG
NOS-F	CGCGCGCGGTGTCATCTATG
1300-R	CTCGTATGTTGTGTGGAATTG
AtGL2-F	AGTTAGGGTTCAGTTGCATG
AtGL2-R	AGTTATAGTAGCTGGTAACAG
GL2-sg97-F	GATTGTCGGAGCATGAAGCCTGCA
GL2-sg97-R	AAACTGCAGGCTTCATGCTCCGAC

YAOT-F	ATGCGGATCCGCATAAGTATTTCATTGGGAT
YAOT-R	ATGCGAATTCCCGGCGAATCGAGGTATGGCTAC
YAOT-F1	CTACTACTTGAATGAATTGTCTTAAGTCAAGCTTAG
YAOT-R1	CAATTCATTCAAGTAGTAGGCTCT

Methods S1

Materials: Backbone vectors: pAtU6-sgR(Amp+); pAtU3b- sgR (Amp+); pAt7SL- sgR (Amp+); pCDC45-Cas9-1300(Kan+).

Restriction enzyme: *Bbs*I; *Hind*III; *Sal*I; *Xma*I, T4 DNA Ligase, ClonExpress MultiS One Step Cloning Kit (Catlog #C113, http://en.vazyme.com/products_detail/productId=67.html).

Methods

Design and synthesize two oliogs of the sgRNA

For pAtU6-sgR:

```
CF: 5′-GATTGNNNNNNNNNNNNNNNNNNNN     -3′
CR:3′-      CNNNNNNNNNNNNNNNNNNNNCAAA-5′
```

For pAtU3b-sgR:

```
CF: 5′-GGTCGNNNNNNNNNNNNNNNNNNNN     -3′
CR: 3′-     CNNNNNNNNNNNNNNNNNNNNCAAA-5′
```

For pAt7SL-sgR:

```
CF: 5′-TTACGNNNNNNNNNNNNNNNNNNNNN-3′
CR: 3′-     CNNNNNNNNNNNNNNNNNNNNCAAA-5′
```

Oligo annealing and cloning into the corresponding backbone vectors.

1. Digest 1μg of pAtu6-sgR, pAtU3b-sgR, pAt7SL-sgR with *Bbs*I for 2h at 37℃.

   1μg pAtu6-sgR or pAtU3b-sgR or pAt7SL-sgR

   1μL *Bbs*I -HF (20 U/μL ,NEB)

   2μL 10 x CutSmart Buffer

   Up to 20μL ddH2O

2. Gel purified digested pAtu6-sgR, pAtU3b-sgR, pAt7SL-sgR using Gel Extraction Kit and elute in EB solution.

3. Phosphorylate and anneal each pair of oligos:

1 µL oligo 1 (100mM)

1 µL oligo 2 (100mM)

1 µL 10 x T4 Ligation Buffer

0.5 µL T4 Polynucleotide Kinase (10 U/µL ,NEB)

Up to10µL ddH2O

Anneal in a thermocycler using the following parameters:

37℃ 30 min

95℃ 5 min and then ramp down to 25℃ at 5℃/min

4. Set up ligation reaction and incubate at 22℃ for 30 min:

X µL *Bbs*I digested pAtu6-sgR, pAtU3b-sgR or pAt7SL-sgR from step 2

1 µL annealed oligo duplex from step 3

1 µL 10 x T4 ligase Buffer

1 µL T4 DNA Ligase (400 U/µL , NEB)

Up to10µL ddH2O

5. Transformation and identify postive clone by Colony PCR (primer M13F+CR or CF+M13R).

SgRNA cloned into binary vector(pCDC45-Cas9-1300)

1, Amplify the sgRNA-cassette (pAtU6-sgRNA, pAtU3b-sgRNA, pAt7SL-sgRNA) with corresponding primer pairs and purified the PCR products.

Primers:	pAtU6-sgRNA	VU6-1F+VU6-R
	pAtU3b-sgRNA	VU3-F+VU3-R
	pAt7SL-sgRNA	V7SL-F+V7SL-1R

2, Digest 1µg of pCDC45-Cas9-1300 with *Hind*III and *Sal*I and purified the constructs.

3, Set up Homologous recombination reaction and incubate at 37℃ for 30 min:

4 µL	5x CE MutiS Buffer (Vazyme)
2 µL	Exnase MutiS (Vazyme)
X µL	Fragement 1 (step 1)
X µL	Fragement 2 (step 1)
X µL	Fragement 3 (step 1)
X µL	digested vector (step 2)

Up to 20 µL ddH_2O

Transformation and select the correct clone by Colony PCR (1300-F+CDC45-R) for the 3sgRNA-pCDC45-Cas9-1300.

4, Amplify the sgRNA-cassette (pAtU6-sgRNA, pAtU3b-sgRNA, pAt7SL-sgRNA) with corresponding primer pairs and purified the PCR products.

Primers: pAtU6-sgRNA VU6-2F+VU6-R

pAtU3b-sgRNA VU3-F+VU3-R

pAt7SL-sgRNA V7SL-F+V7SL-2R

5, Digested 1µg of 3sgRNA-CDC45-Cas9-1300 with *Xma*I and purified the construct.

6, Set up Homologous recombination reaction and incubate at 37℃ for 30 min:

4 µL   5x CE MutiS Buffer (Vazyme)

2 µL   Exnase MutiS (Vazyme)

X µL   Fragement 1(step 4)

X µL   Fragement 2 (step 4)

X µL   Fragement 3 (step 4)

X µL   digested vector(step 5)

Up to 20 µL ddH_2O

Transformation and select the correct clone by Colony PCR (NOS-F+1300-R) for the 6sgRNA-pCDC45-Cas9-1300

List of primers used for construction of the multi-pCDC45-Cas9-1300.

	**6sgRNA** PRIMER	**5sgRNA** PRIMER	**4sgRNA** PRIMER	**3sgRNA** PRIMER	**2sgRNA** PRIMER	**1sgRNA** PRIMER	
AtU6-sgRNA	VU6-1F+VU6-R	VU6-1F+VU6-R	VU6-1F+VU6-R	VU6-1F+VU6-R	VU6-1F+VU6-R	VU6-1F+VU6-1R	step1
AtU3b-sgRNA	VU3-F+VU3-R	VU3-F+VU3-R	VU3-F+VU3-1R	VU3-F+VU3-R	VU3-F+VU3-1R		step1
At7SL-sgRNA	V7SL-F+V7SL-1R	V7SL-F+V7SL-1R		V7SL-F+V7SL-1R			step1
AtU6-sgRNA	VU6-2F+VU6-R	VU6-2F+VU6-R	VU6-2F+VU6-R				step4
AtU3b-sgRNA	VU3-F+VU3-R	VU3-F+VU3-2R	VU3-F+VU3-2R				step4
At7SL-sgRNA	V7SL-F+V7SL-2R						step4

Primer list

VU6-1F AACGACGGCCAGTGCCAAGCTTCATTCGGAGTTTTTGTATCTTG

VU6-R GTTATCCATCACAGGCTCGAGCCATTTGTCTGCAGAATTGGC

VU6-1R CTTTATCATCAGGAGGTCGACCCATTTGTCTGCAGAATTGGC

VU3-F CTCGAGCCTGTGATGGATAAC

VU3-R GCCAATTTACCAGCATCTAGACCATTTGTCTGCAGAATTGGC

VU3-1R CTTTATCATCAGGAGGTCGACCCATTTGTCTGCAGAATTGGC

V7SL-F TCTAGATGCTGGTAAATTGGC

V7SL-1R CTTTATCATCAGGAGGTCGACGCCATTTGTCTGCAGAATTGGC

VU6-2F ATGTTACTAGATCGGCCCGGGCATTCGGAGTTTTTGTATC

VU3-2R AATTCGAGCTCGGTACCCGGGCCATTTGTCTGCAGAATTGGC

V7SL-2R AATTCGAGCTCGGTACCCGGGGCCATTTGTCTGCAGAATTG

1300-F GGCGATTAAGTTGGGTAACG

CDC45-R GACATTGGTCTAGAGTTACAG

NOS-F CGCGCGCGGTGTCATCTATG

1300-R CTCGTATGTTGTGTGGAA

PROMOTER SEQUENCE

pCDC45

ctcctgatgataaaggtgggagtgtcattatttatattaaaaaagtaagaataatatcatatggaaaaaaaaatttggtctgttacaaaagtaaagcctatg

gtttataaatccttgacaaagaaaataaaaataaaaaatcaaattaggaaaaacaaaaatcggcatgatcatggctaattaggttttgatcatcttctagatt

cttaatagtttttttgcttgtagtagtaatgacgaacacattttgcacactgtaactctagaccaatgtcgtaaaatactttacaatttgaggtaatgatacaa

atataaagattaaagcaatgctaatatattgaatcaaagcaaatattaacattaataaaatgttttgaaagataattgttttgacttttgactagtcgtaagtag

caatttactagagatgaggaattgtataagattaaggatgtatttcactatgagattttagagttagctttgaagagtgtcaaattggaatatcatgttggtgg

aaaattattatgctttgaaggttggaataagcattggaaagccgatgatgttgagatatacatttacaatggtctcgtaatcaggtgctaaatattgcaagg

acatgcgttgtgtaattatcattgatttcacaataacccatggggtaaatgtggtaatttgcaaaagtagtaagggagtggaatttggtaattgtggataca

ttgctgtcaatgttgaaataatgctaactttggttggtgctgcaaaaaaaaaggtcagccaaatatgtgtagaacgaagccgcaatatctactacaagatt

gctactctatagatgttgaatggggttcccaaccacttataaaagtggatcaagatcaagaaaaggagaacatgaagtttaaggtttgtgtgtgtggatat

tccttcatttgaatgtggcttcctagatgttgagttcatgtttttgttttcttacatcattaggtagcaactgattgttttggtagcttgtttgctcatatatatcaaa

attctgatagaattggaaacacatgtttgttgttatggtattagcatatgttactcattatacacacaagtacataagtttattgtgacaaaagtgctgatattat

gtgaaggttatgtagagaatccgtggagggttggtgacgttaatgactgttgtggatgtgttaaatctttggtaatgtttctttccagctttaattacatcctc

aaatgccaatgattcaatagtagtgtaatctcctgcacaaccgcaagacacctgctcaaaaccaaaacaaacaaaaattgttttcgaattgcttttaacttt

ggtttctcgtacaaaaaaactaatacaaaggtgcatgagtagtagtaactgaataaataataagtgcaaatagttgacttaaaataagaagttgaaagag

ttgaatgatttcatctcactttttaccatttgagataaataagtttgaaaattgattcaacttattcaaccacaaaaattagaaaactaaaccaattataaaaaa

cgaattgttcaaccctttcatctactattttggagaggaattgattaaatgatgttcaacttttttttcaaactcaaaattttcctatcatttatatatctagcaattc

atttgcaactgaaatgttttttcgtgtcaaactctaggtttgaaacccgtatacatcaccaacaataactagagtttgaaatcaaataatatttttcatatata

aaacaatttaaaatttcttttagataattaataaaaaatgtatgacacacagacacagctggttgatttgtttatttgaatttttgactaggattaagaacattaa

tcatcaaccgttgattatataagcaagttgaagaaaaggcacggttcagattcacctcttctcatatgatgcgttacataagatcctttccctctttccctcat

tttcaggttcagcgccatcgtcaaaaacttttggcggtcgatcaaaaaatttcgaaatttgaagatttcgccggcaaatacacaaacccgaaaatgaata

acacttcaaaattttcattaccagaagaaagaaatcaaataccttcagatctctatcttcctcattcacacaccctctctctcttctccttttctctcttctcctttt

ctctatctccctctttgttccgttcgcatcctctaatcatcgtcaacaagccgacgaagagagaaacgaatccaaagttcgttacttgaaagctacccaga

agaattcaaatctcaggtacttttcctgtggatttgatctgggcactgcttattagggatttgattggatctacaaaattctgccttctgggtgattcaatttca

cggaa

pDMC1

cagggaatgttccaatataagacactttaaacgtaagtttagacaatatagacactttccaagttagaggcacttttccttctttttgaaggaaaacttgactt

ttatacctcttaactaaacaatcgaaaacaataactaaatatatatcttaaccaaacaattaaaaaaaataaaagaatttagatacgtagttattaatatagacc

attagattgaaaaataaaaattaagatctatggctgagattaaagacaataaatggattaatttttgatgttaaaatctgattagaaaaaggtatttctcttcg

tctctagaactaaatctctctctctaaaaaaacaatcgtttctccctttctccttcctgaagatcgtttttcataaatccatagtagtttaaaaacgaagcaga

gagatgttgaaaatcgtttctcatgaaattaatcgattattctctgtgaagttctttaatccacacaactttcctcatgaacatgataatagtagtaaatggag

gtttttcctatggttactctagacgaaggaggatctccttgtgttggacaggtttgtgatttctttccatggattaaaaaaatttgattgtttgtttatgatgaac

gattctttggctacggaagagtgtcatggagttctggcgaattctttggctatgtttggtgatttcgtttttaatcaagttgggaatcaataggaaacaacta

agcatacaacatagattagaagagatatcaagatggatctaatttaagtaagatttggcgactaattctagatgattagggttatttgtgatttattacaagg

catttgtgttctcattgatttggcgagtaattctgtatgactagggttatttgtgttttcttaaaaagaatttgtgttcttgttgaaatcttgttcattggaattatttg

tgtttggtaaatcttcattggtggctaaggatgtgtttgtagctcttacggcgtttgttattggtgatgtccattatggatggcaaattatggatggcacattat

ggatgatgaatcatggatgacatattatggatgacgcatcatggattgtatattatggattgatatggtgagatttgtaaatcttttggtcttacatgttaaga

gtaaaagatgaagaattggagaagcatgtctaacatcctaaaaacaagctatatgcggttgatttgctacaaataatttttggtatccataataacaaatc

catttaaatatatccattcagaaacctttctactgatccgtatccattctatataccatgtcaataataataggagattcgattaaccgtgttttgtaaagaaac

caaagttccatgtccataaggttttgaaggtggaggtctctgcaaactgaaaaaaaaatcaacaaacaatttttggtgtccataataacgaatccatttaa

atatatccattcggaaacctttttactgatctatatccattttatataacatgtccatgataacaggagattcgattaactgaaatctcgatgctacgtagatga

aacgagtttgacacatgagagagagcaaaaatcaaatcaaaccgccattgttgaagaagaagaagtttcttctcatttttacaaagatgaagagagag

agaggtgaagagagagagagagatgaagagagagagagagagaaagagagagatgaagagagagagagaaagagagaaaacgtgggttaa

gataatattttagttaagagggtattttagtaaaaaaacataaaaaagtgcctaatcttttgaaagtgcctaaacacagaaatagttttaaaaaagtgtttaa

gagtgtaatattctcttttttcacctagattccttctattgaccgtcgatagacggatgataactatgacgtggcattatcgcagccatcaaacaaagtcat

gtataacaaagaagagcacacaaacgaaaacaaattcagttgcggaacccaaattcaaatcaacggaattagaatcacgctttcaattccgtaacccg

ccattaaaaaccttgaaccctcgaagcaaatcgagcaaagattttcaaatttcgaatttcaaaattctatctctctcactcttccaagcttagagactcttag

agcgagaaa

pSPO11

gcatttgcagctcgtctggttcgagccttgtgacgttaaaagcaagctgtatttgtctctgctgagagtttggatgcaattagacgaagaggataaatgaa

aacaccttctcttttattcgtagagttgggttttatgagagaagtgtaaacttgtagagatgctgctaattgtatgttgatatgtttaaagaaaatgcctcgcc

gttttgccagagctagaaagtagaaacggcggctcgtgtaagttctaacaaaagagtagtcgtctgttagcaacccaaatcgggcttttaaaggcccat

attttggtcggcctgaatccaaagttaaaaattcaatcatgcaaaacggacaacattataactgaattgaaaatatctctcttatcattattttagctttgtacg

atttactatttatgtcagaaaatgtttcattctttgacttttacgacaactttacaaaaaaaaaaaatgttcatacaagtcacagaacaaagtaacataaaaatct

taaaaatatattatcattttcgttagaagaaaaacagtataattttcaaacgcgtttaaaaacaaaataacttgagattttgacgttatccttgtcacagagaat

ctttcctatttctttacgtatagacactcagaggaaagacagagagagagttagcattgtactatctctcttttagatatatctctatctctcttcactccatcttt

ctcgtgttaacacaacagtcacagactcacagatctttgtcggtgatcggagatggagttccgggagaagcttttgaagttcaagttgcaaatcgtgttt

gcctttgttttatctctcctcaccgtagctctcgtcgcttattctcccggtttcctcaccgttctatcttacttctggccactttttctctccacggctctctttctcg

ctgccgttttcttcttcgcacgcacctccgatctcccggtttcgtctaccatcatcccaggcgaaggcgcaggcttaaaagtggcggcggaagggattc

ttgactatgtcgtcggcggacaacacgaagacattctctttgatagcttcaccaaacttgactaagtatgagacctctctgttcttaattccttaagtatttcc

gttaattagttgttcctttttttaatactagagaaaataaaattacaaaatgtaatattttcacgaggaataaaatcttgcttaagtttatgcttctatattacttatttt

aatcttatgtttctgtttaggcgcgaggcttcttttctttaggtatctctcattagtttatacgttacaagattacacattgcattgatatatgattttactttcaaaa

aaaatttaaatgttgaacgaccttaaaatccgtgcgctttacttttaaatctttccgaatgaaacccaaccaccgacgcgacagcttttcatttgtttactaatt

aaacggaacaaagcaatttaaatgttatcagtgttgtgttttagttagaaatactataaataatacaagatgcctcttttgttttagaactttttttggtatcatga

attcgttatcggataaaatacgttaaagctaagtagtatattcttagattatatataaattaggtgagcaatctatcatagagaagaaatgattaaaataagtg

tatggcataatagattcgaacattttggtatgttcctcacgtgtccgagcataaagcttatgaacaaatctcgaccactagaagtctagaactaattctaatc

tagtttaccatacacattaattatttcaaatttagtgcacatgattattttatattggcatcactaaaatgtctccaagtctgaataaatcgatttatattggtttga

ttgttatcatgaatctttttcacaatcactttgcctagtttcggatcgggcctaaaagccaacggcccaacgtgtttaaaaacacatgcgtttttaaaatgac

acgtcagttacgtatcgggcctaaatgccaatggcccaacgtcatggaaagtaggaactaaacgacacgcatgtttaaattcgaaggttttaacggca

agcttttcgcacccatcccttttcttcccgtctacaaaaattcgtcgaccattgcatttttcagttttgaaactcgaaagag
